# Comment on “Construct validation of machine learning models for predicting surgical site infection risk following ankle fracture surgery”

**DOI:** 10.1097/JS9.0000000000004932

**Published:** 2026-06-03

**Authors:** Yu Fu

**Affiliations:** Reproductive Medicine Center, Hainan Women and Children’s Medical Center, Haikou, China

*To the Editor,*

Zhang *et al* report a single-center retrospective cohort (n = 509) developing machine-learning (ML) models to predict 30-day surgical site infection (SSI) after ankle fracture open reduction and internal fixation (ORIF), with an SSI rate of 10.0% and best performance from a gradient boosting machine (area under the curve [AUC] 0.919)[1]. The identified top contributors – hypoalbuminemia, open injury, diabetes, hypertension, and longer operative duration – are clinically intuitive and align with established ankle ORIF complication patterns where wound infection is typically much lower in broad administrative cohorts, highlighting how case-mix and surveillance intensity can shift baseline risk and calibration^[^1,2^]^.

Two aspects make this study particularly useful for practice. First, the authors combine discrimination with calibration and decision-curve analysis, which is essential when models are intended to trigger actions rather than merely “predict”[1]. Second, the SHAP-based interpretation offers a clinician-friendly bridge from model output to modifiable perioperative domains (nutritional optimization, glycemic control, soft-tissue strategy for open fractures, operative efficiency)[1].

However, several points should shape how readers interpret readiness for deployment. The relatively high SSI prevalence in this dataset (10%) compared with prior large ORIF series (≈1–2% wound infection) implies that absolute-risk thresholds learned here may not transport to other hospitals; external and, ideally, temporal validation are therefore pivotal before using fixed cutoffs to intensify antibiotics or escalate invasive monitoring pathways^[^1,2^]^. In addition, rare-event prediction often yields limited precision even with high AUC, so “risk-guided stewardship” (optimization and follow-up intensity) may be safer than default antimicrobial escalation for all flagged patients.

From a reporting perspective, mapping the work explicitly to contemporary prediction-model guidance would further strengthen credibility and reproducibility – particularly clear outcome labeling, missing-data handling, intended-use setting, and the clinical decision consequences of false positives/negatives, as emphasized by TRIPOD + AI and PROBAST^[^3,4^]^. We prepared this commentary in accordance with the TITAN 2025 guidelines to ensure transparent and responsible reporting[5].

Overall, Zhang *et al* provide a promising, interpretable ML framework for SSI risk stratification after ankle ORIF. The next step is multicenter external validation with recalibration and impact analysis to show that integrating the model into perioperative workflows measurably reduces SSI without unintended harms[1].

## Data Availability

No data were used in this Letter to the Editor.
